# Models of Metaplasticity: A Review of Concepts

**DOI:** 10.3389/fncom.2015.00138

**Published:** 2015-11-10

**Authors:** Pierre Yger, Matthieu Gilson

**Affiliations:** ^1^Sorbonne Université, UPMC Univ Paris06 UMRS968Paris, France; ^2^Institut de la Vision, INSERM, U968, Centre National de la Recherche Scientifique, UMR7210Paris, France; ^3^Computational Neurosciences Group, Departament de Tecnologies de la Informació i les Comunicacions, Universitat Pompeu FabraBarcelona, Spain

**Keywords:** synaptic plasticity, metaplasticity, Hebbian learning, homeostasis, STDP

## Abstract

Part of hippocampal and cortical plasticity is characterized by synaptic modifications that depend on the joint activity of the pre- and post-synaptic neurons. To which extent those changes are determined by the exact timing and the average firing rates is still a matter of debate; this may vary from brain area to brain area, as well as across neuron types. However, it has been robustly observed both *in vitro* and *in vivo* that plasticity itself slowly adapts as a function of the dynamical context, a phenomena commonly referred to as metaplasticity. An alternative concept considers the regulation of groups of synapses with an objective at the neuronal level, for example, maintaining a given average firing rate. In that case, the change in the strength of a particular synapse of the group (e.g., due to Hebbian learning) affects others' strengths, which has been coined as heterosynaptic plasticity. Classically, Hebbian synaptic plasticity is paired in neuron network models with such mechanisms in order to stabilize the activity and/or the weight structure. Here, we present an oriented review that brings together various concepts from heterosynaptic plasticity to metaplasticity, and show how they interact with Hebbian-type learning. We focus on approaches that are nowadays used to incorporate those mechanisms to state-of-the-art models of spiking plasticity inspired by experimental observations in the hippocampus and cortex. Making the point that metaplasticity is an ubiquitous mechanism acting on top of classical Hebbian learning and promoting the stability of neural function over multiple timescales, we stress the need for incorporating it as a key element in the framework of plasticity models. Bridging theoretical and experimental results suggests a more functional role for metaplasticity mechanisms than simply stabilizing neural activity.

## 1. Introduction

The brain is made of billions of neurons able to efficiently process the huge flow of information impinging continuously on sensory modalities, extracting relevant data, and producing appropriately timed responses. Even during development (Corlew et al., 2007; Wang et al., 2012) or when lesioned (Young et al., 2007; Beck and Yaari, 2008), the brain has the striking capability to adapt in order to maintain the stability of neural functions. Importantly, this slow adaptation, acting at a timescale of hours or days (Turrigiano and Nelson, 2000; Davis, 2006) is performed in conjunction with fast changes often observed in the so called Hebbian learning (Hebb, 1949). Understanding the mechanisms leading to the dynamical organization of neuronal network via the fine interactions of those two competing processes is therefore a crucial step toward analyzing the stability of the computations performed by cerebral activity.

Following the seminal idea that neurons firing together should wire together (Hebb, 1949), numerous experimental studies have been conducted to unravel part of the links between plasticity and neuronal activity. Nowadays, this so-called Hebbian form of plasticity in the brain has been characterized experimentally in many areas, involving multiple but still misunderstood molecular pathways (see Abbott and Nelson, 2000; Caporale and Dan, 2008, for reviews). While it is commonly assumed that NMDA receptors are the primary actors in long-term potentiation, or LTP (Feldman, 2012), the biochemical pathways for long-term depression (LTD) seem to differ in cortex and in hippocampus (Wang et al., 2005; Bender et al., 2006; Nevian and Sakmann, 2006). In controlled *in vitro* experiments, it has also been shown that LTP and LTD depend on the precise timing of pre- and post-synaptic spikes (Markram et al., 1997; Bi and Poo, 1998), leading to the concept of timing-LTP/LTD or spike-timing-dependent plasticity (STDP).

By acting independently at each synapse without spatial or temporal crosstalk among synapses, Hebbian learning is a form of homosynaptic plasticity that is intrinsically unstable. In point of fact, provided synapses are reinforced when both the pre- and post-synaptic neurons are active, nothing prevents the synapses from strengthening themselves boundlessly, which causes the post-synaptic activity to explode (Rochester et al., 1956; von der Malsburg, 1973; Miller, 1996). While this instability can be avoided by artificially imposing hard boundaries onto the synaptic weights, several learning models came with intrinsic mechanisms regulating the synaptic efficacies (Bienenstock et al., 1982; Oja, 1982) in order to solve this issue in a less fine-tuned manner.

The present paper reviews such mechanisms that aim to tame the positive feedback provided by Hebbian plasticity. In the biology, some homeostatic mechanisms can be viewed as independent from the Hebbian learning that they counterbalance. For example, the sum of synaptic strengths may be up or down regulated to maintain the average post-synaptic firing rate; see Vitureira and Goda (2013) for a review of the biophysics of such mechanisms. In contrast, other processes directly modulate the learning rule itself as a function of the dynamical context, which is referred to as metaplasticity. This concept is the plasticity of the synaptic plasticity itself (Abraham and Bear, 1996; Abraham, 2008), and it is tightly related to the notion of homeostasis (O'Leary and Wyllie, 2011). To ensure the overall stability of the neuronal system, a key role for metaplasticity is to regulate the synaptic update rules in terms of the past history of the activity at the whole neuronal level. Many experiments have demonstrated metaplasticity using distinct protocols (Abraham, 2008). Quite often, it also involves some form of heterosynaptic plasticity, in the sense that the local changes affecting a particular synapse onto a post-synaptic neuron influence the plasticity for neighboring synapses.

The study of the dynamical implications of the interaction between homeostatic mechanisms and Hebbian plasticity requires the integration of experimental data in model studies (Marder and Goaillard, 2006). From a modeler's point of view, interactions between Hebbian learning and its regulating counterpart, either by homeostatic mechanisms or by metaplasticity, is problematic. The principal reason being that those two distinct forms of plasticity do not act on similar timescales. Following experimental results, it is commonly assumed that synaptic changes triggered by Hebbian plasticity protocols are rather fast (Bliss and Lomo, 1973; Sjöström et al., 2001, 2003; Wang et al., 2005), occurring in the timescale of minutes or faster, while metaplasticity or homeostatic changes are much slower (Abraham and Bear, 1996), in the order of days. The present paper provides a theoretical framework to analyze the interaction between Hebbian and homeostatic plasticities at different timescales. In this way it gives an overarching view of different methods used in the literature to solve the above-mentioned instability issue of Hebbian plasticity. Maintaining the stability only being one of the requirements for proper behavior, we will discuss how homeostatic constraints can also be used to adjust the function implemented by the neural circuits.

## 2. The apparent antagonism between Hebbian and homeostatic plasticity

### 2.1. Two divergent goals

As it has already been observed (Turrigiano and Nelson, 2000; Watt and Desai, 2010; Vitureira and Goda, 2013), Hebbian and homeostatic plasticities are two apparently opposing processes, which compete at the synaptic level to fulfill different goals. Hebbian learning promotes strong or synchronous firing among neurons, which is hypothesized to be a building block for memory storage (Nabavi et al., 2014). In contrast, homeostatic processes counterbalance such intense spiking activity to maintain the global stability in neuronal networks (Turrigiano and Nelson, 2000; Turrigiano, 2008; Pozo and Goda, 2010). Several types of homeostatic processes have been observed at the neuronal level in many brain areas, such as synaptic scaling (Turrigiano et al., 1998) and intrinsic plasticity (Zhang and Linden, 2003).

It has been long known that Hebbian plasticity *alone* is intrinsically unstable (Rochester et al., 1956; von der Malsburg, 1973; Miller, 1996). The entrainment between synapses often force all to grow boundlessly or to a maximal set value; in other cases, they may all become silent. To circumvent these issues of traditional rate-based Hebbian learning, weight normalization can be introduced to prevent the runaway of synapses (Oja, 1982; Miller, 1996). In the context of spiking activity, STDP has been termed “temporally Hebbian” when it promotes synchronous firing. Weight-dependent STDP update rules, which induces more LTD than LTP for strong synapses, provide a fixed point in the learning dynamics (van Rossum et al., 2000; Gütig et al., 2003). Although this ensures some stability, it may dramatically change the weight distribution from being bimodal to being unimodal. In the case of a narrow unimodal weight distribution, competition induced by STDP among synapses is weakened between pathways with distinct characteristics (e.g., rate, correlation), which is not functionally interesting. For weight-dependent STDP, this trade-off compromise is only fulfilled in a given parameter range. In recurrent networks especially, the synaptic specialization by competition may be severely impaired without fine tuning (Morrison et al., 2007; Gilson and Fukai, 2011).

### 2.2. Two different timescales

**Figure 1:**
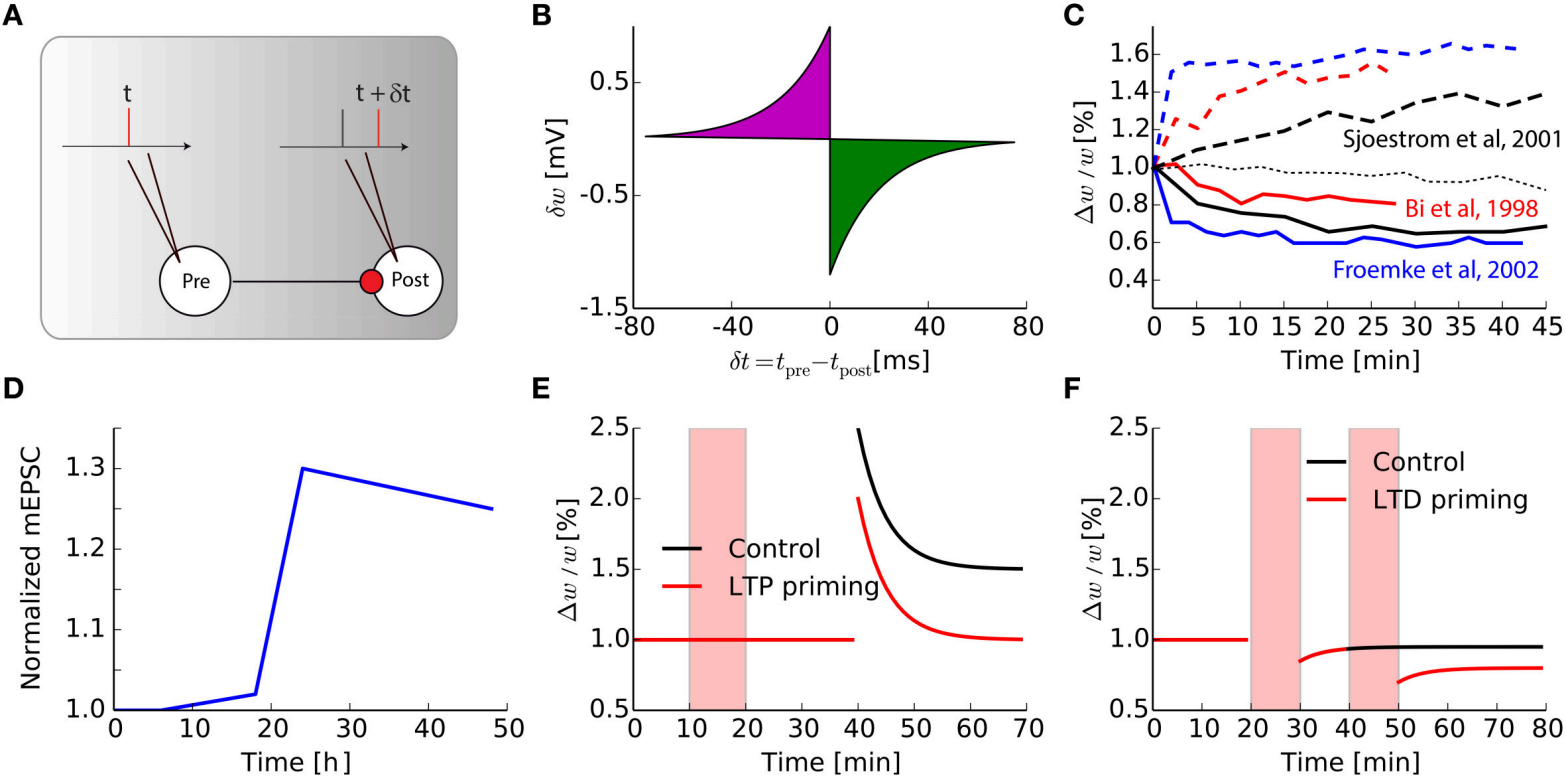
**Intrinsic timescale of Hebbian learning**. **(A)** The classical STDP pairing protocols widely used in the literature. **(B)** Synaptic modification for one pair of pre- and post-synaptic spikes, as a function of their relative timing. **(C)** Evolution as a function of time of a single synaptic weight, after an STDP protocol, for various papers taken from the literature, both for LTP of LTD protocols [dash-dotted thin black line is the null-line for Sjöström et al. (2001)]. **(D)** Adapted from Keck et al. (2013), Normalized mEPSC amplitude in a layer 5 cell in the mice visual cortex following a lesion in the retina. **(E)** Adapted from Huang et al. (1992), Prior synaptic activity triggered during the red shaded area (LTP priming, red curve) reduces LTP in CA1 hippocampus compared to control without pre-activation (black curve). **(F)** Adapted from Mockett et al. (2002), Low frequency stimulation (LFS, red shaded areas) influences non-linearly the amount of LTD in CA1 hippocampus: black curve, (control with only one LFS), red curve (two consecutive LFS).

Most of the plasticity protocols performed *in vitro* are based on either input stimulation at a high/low frequency leading to LTP/LTD (Bliss and Lomo, 1973) or STDP-type pairings of pre-post spikes (Markram et al., 1997; Bi and Poo, 1998; Sjöström et al., 2001; Froemke and Dan, 2002; Wang et al., 2005). The typical protocol used in cortical or hippocampal slices to elicit STDP *in vitro* using spike pairs is represented in Figure 1A: a spike is triggered at the pre-synaptic neuron and another at the post-synaptic neuron with time difference δ*t* = *t*_pre_ − *t*_post_. This pairing is repeated approximately 60 times with frequency *f*_pairing_ = 1 Hz in order to see a robust change in the weight: it has been shown that after an induction phase, the total weight change evolves non-linearly up to a saturation plateau, at around 60–100 pairings (Froemke et al., 2006), which corresponds to the number of protocol repetitions in most studies.

For modelers, this STDP protocol leads to the simplified view of the time-difference window in Figure 1B, where a single pre spike followed by a post spike will trigger LTP, whereas post followed by pre causes LTD. This is clearly an over-simplification of a much more complex phenomenon. Just to mention some limitations of this simplified view, it has been shown that if the frequency *f*_pairing_ of the pairing is changed, the typical STDP curve with LTP for δ*t* < 0 and LTD for δ*t* > 0 is dramatically modified (Sjöström et al., 2001). Depression is only visible for low frequency pairings, when pairings are performed with δ*t* < 0 and *f*_pairing_ < 20 Hz. For *f*_pairing_ > 20 Hz, however, synapses undergo LTP irrespective of the sign for δ*t*. Moreover, several *in vitro* studies on cortical pyramidal neurons showed that the canonical shape of the STDP curve for such pre-post pairings strongly depends of the position of the synapse along the dendritic tree (Froemke et al., 2005; Letzkus et al., 2006; Kampa et al., 2007), as well as the post-synaptic voltage (Artola et al., 1990). Those experimental findings led to the refinements of initial STDP models based on the curve, in order to incorporate the observed effects for triplets of spikes, spike bursts, clamping the post-synaptic membrane potential and so on (Pfister and Gerstner, 2006; Clopath et al., 2010; El Boustani et al., 2012; Graupner and Brunel, 2012; Yger and Harris, 2013).

Despite those efforts, there is a point that is almost never considered: STDP changes are not instantaneous. In most experiments, when plasticity protocols are performed, the resulting weight is recorded up to 30 min later. The curve in Figure 1B corresponds to the corresponding weight change divided by the number of pairings. In models of classical (van Rossum et al., 2000; Song and Abbott, 2001) and weight-dependent (van Rossum et al., 2000; Gütig et al., 2003; Morrison et al., 2007; Gilson and Fukai, 2011) STDP, its final value is the results of additive instantaneous and independent weight updates following each pairing. In fact, even elaborate models consider the linear summation of weight updates, even when contributions are restricted to neighboring spikes (Burkitt et al., 2004). Only a few attempts have been done to change this property that is convenient for theory, such as probabilistic models of STDP (Appleby and Elliott, 2005). By re-examining the weight traces found in the STDP literature (Bi and Poo, 1998; Sjöström et al., 2001; Froemke and Dan, 2002; Froemke et al., 2006) and reproduced in Figure 1C, it can be seen that the weights actually evolve continuously *in vitro*. Therefore, plasticity should better be seen as a phenomenon that is triggered by a stimulation event and evolves toward a new equilibrium with a time constant τ_Hebb_ ≃ 10 min.

Now considering that Hebbian plasticity induces such a transient synaptic change, the question arises about its interaction with homeostatic plasticity. Those processes, either intrinsic or synaptic, are assumed to be much slower. For example synaptic scaling, one of the numerous mechanisms of homeostasis, takes place *in vitro* with a time constant τ_homeo_ of the order of a day (Turrigiano and Nelson, 2000), and *in vivo* during the 2–3 days after an abrupt change, as observed for neurons in the visual cortex following visual deprivation (Hengen et al., 2013; Keck et al., 2013). Figure 1D, adapted from Keck et al. (2013), shows the amplitude of miniature EPSC in V1 neurons after a bilateral lesion in the adult retina: after an initial period of about a day, amplitudes are scaled up to compensate for the reduced inputs. Together, these results stress the fact that Hebbian and homeostatic processes have distinct timescales. Understanding the biological mechanisms responsible for those changes at the molecular level is necessary to gain a better insight on the interaction between them, especially *in vivo* where synapses are constantly bombarded by spikes.

### 2.3. Primings as an evidence for metaplasticity

Although on a first approximation it may appear that τ_homeo_ ≫ τ_Hebb_, several experiments show that those two timescales may be more interleaved. In hippocampal slices, it has been shown in so-called priming experiments that the activation of a synapse before its reactivation modulates the plasticity triggered later at that particular synapse (see Figures 1E,F). In Figure 1E that is adapted from Huang et al. (1992), weak tetanic priming stimulations can reduce the amount of LTP obtained during a strong subsequent tetanic stimulation; note that the effects last more than 1 h. On the contrary, the LTD pathway seems to be facilitated when the synapse is preactivated a few hours before the plasticity protocol (Christie and Abraham, 1992; Wang, 1998; Mockett et al., 2002). This is illustrated in Figure 1F, adapted from Mockett et al. (2002), where the effect lasts at least 2 h. Those primings experiments suggest the existence of long-lasting regulation mechanisms, acting over large time constants, which counteracts the effect of Hebbian learning. This modulation of the Hebbian plasticity by preactivation of the synaptic pathway is a direct application of the so-called metaplasticity (Abraham and Bear, 1996), i.e., the plasticity of the learning rules themselves.

## 3. Mathematical formalism

To formally study the interactions between Hebbian and homeostatic plasticity, we use the following mathematical formalism. We consider a Poisson neuron (Kempter et al., 1999) with *N* synapses indexed by *i*, corresponding to the input firing rates *r*_*i*_; for STDP examples, we also define the input cross-covariances *c*_*ij*_ between neurons *i* and *j*. The equations for the output firing rate *r*_post_ and pre-post covariances *c*_*i*−post_ between synapse *i* and the post-synaptic spike train in a feedforward scenario are given by

(1) $$\begin{array}{l} \;r_{\text{post}}=\sum_{1\leq j\leq N}w_{j}\;r_{j}\\ c_{i\text{-post}}=\sum_{1\leq j\leq N}w_{j}\;c_{ij} \end{array}$$

In order to compare several learning rules in the context of metaplasticity, we consider the following general equations for the evolution of a given weight *w*_*i*_ and a modulation parameter θ:

(2) $$\begin{array}{l} {\dot{w}}_{i}=\frac{1}{\tau_{\text{Hebb}}}\Phi (w_{i},r_{i},r_{\text{post}},c_{i\text{-post}},\theta )\\ \dot{\theta }=\frac{1}{\tau_{\text{homeo}}}[\Psi (r_{\text{post}})-\theta ] \end{array}$$

The motivation for these expressions is to model the two timescales explicitly, as previously done for the BCM rule (Bienenstock et al., 1982) and for a extension of the triplet STDP rule (Zenke et al., 2013): τ_Hebb_ and τ_homeo_ are the two time constants at which both Hebbian and homeostatic changes are propagated onto the synapses. The Hebbian plasticity update is embodied in Φ, which also depends on *r*_pre_, *c*_pre−post_, etc. The parameter θ is global for all synapses of a neuron and interacts or modulates the corresponding weight updates. Typically, it is used to implement a homeostatic mechanism, as we will see for several models of synaptic plasticity that are commonly used in the literature. The present framework could be extended to incorporate other non-linearities in the firing mechanism (e.g., LIF neuron), adaptation or intrinsic plasticity.

### 3.1. Stability analysis for the mean-field dynamical system

Ignoring correlations and inhomogeneities across synapses, we focus on the analysis of the mean weight $\bar{w}=\underset{j}{\sum }w_{j}∕N>0$ with mean input rate *r*_pre_. The rate Equation (1) simply becomes

(3) $$r_{\text{post}}=\bar{w}\;r_{\text{pre}}$$

This allows for an easy comparison of the weight dynamics based on polynomial expressions in $\bar{w}$. Other neuron models usually give more complex mapping between input and output rate/correlations, but the common trend is that they are monotonically increasing function of the weight $\bar{w}$. This property is the cause for the instability of Hebbian learning, as it increases $\bar{w}$ all the more as *r*_post_ is large. Therefore, we will review through the example of the Poisson neuron how stabilization mechanisms interact with the Hebbian component.

In order to examine the stability of the mean-field dynamical system (Equation 2) where *w*_*i*_ is replaced by $\bar{w}$, we consider its Jacobian matrix.

(4) $$\left(\begin{array}{cc} \frac{1}{\tau_{\text{Hebb}}}\left[\frac{\partial \Phi }{\partial w}+\frac{\partial \Phi }{\partial r_{\text{post}}}r_{\text{pre}}\right] & \frac{1}{\tau_{\text{Hebb}}}\frac{\partial \Phi }{\partial \theta }\\ \frac{1}{\tau_{\text{homeo}}}\frac{\partial \Psi }{\partial r_{\text{post}}}r_{\text{pre}} & \frac{-1}{\tau_{\text{homeo}}} \end{array}\right)=\left(\begin{array}{c} a\;b\\ c\;d \end{array}\right)$$

For the top-left term in the Jacobian, we have used the following equality for the feedforward architecture corresponding to Equation (3): $\frac{\partial r_{post}}{\partial w}=r_{pre}$. The eigenvalues of the Jacobian matrix are given by

(5) $$x_{\pm }=\frac{1}{2}(T\pm \sqrt{T^{2}-4D})$$

where *T* = *a* + *d* is the trace and *D* = *ad* − *bc* is the determinant. To ensure stability for this 2-dimensional dynamical system, these eigenvalues must be real negative. This requires that the following relationships are satisfied.

(6) $$\begin{array}{l} \;T\text{ < 0 }\\ 0<4D<T^{2} \end{array}$$

If, however, the discriminant is positive with the trace still negative (*T* < 0 and *T*^2^ < 4*D*) the system exhibits damped oscillations related to the imaginary eigenvalues. With purely imaginary eigenvalues, we may obtain a limit cycle. Finally, when *D* < 0 or *T* > 0, at least one eigenvalue is positive and can lead to an explosion of the mean weight.

### 3.2. Competition between input pathways

Following Kempter et al. (1999); Gütig et al. (2003); Gilson et al. (2009), we can use and rewrite Equation (1) to study the competition between learning weights.

(7) $$r_{\text{post}}=w_{1}r_{1}+w_{2}r_{2}$$

Again ignoring correlations, we obtain the following 3-dimensional learning system.

(8) $$\begin{array}{l} {\dot{w}}_{1}=\frac{1}{\tau_{\text{Hebb}}}\Phi (w_{1},r_{1},r_{\text{post}},\theta )\\ {\dot{w}}_{2}=\frac{1}{\tau_{\text{Hebb}}}\Phi (w_{2},r_{2},r_{\text{post}},\theta )\\ \dot{\theta }=\frac{1}{\tau_{\text{homeo}}}[\Psi (r_{\text{post}})-\theta ] \end{array}$$

Considering the equilibrium for the mean weight $\bar{w}=\left(w_{1}+w_{2}\right)∕2$ to be satisfied, the competition between the two input pathways can be studied for what is called “symmetry breaking,” namely the divergence of *w*_1_ and *w*_2_. This relates to the following differential equation for the weight difference Δ*w* = *w*_1_ − *w*_2_, which quantifies the tendency for splitting

(9) $$\begin{array}{l} \dot{\Delta w}=\frac{1}{\tau_{\text{Hebb}}}\left[\Phi (w_{1},r_{1},r_{\text{post}},\theta )-\Phi (w_{2},r_{2},r_{\text{post}},\theta )\right]\\ \;≃\frac{1}{\tau_{\text{Hebb}}}\frac{\partial \Phi }{\partial w}(\bar{w},{\bar{r}}_{\text{pre}},r_{\text{post}},\theta )\;\Delta w\\ \;+\frac{1}{\tau_{\text{Hebb}}}\frac{\partial \Phi }{\partial r_{\text{pre}}}(\bar{w},{\bar{r}}_{\text{pre}},r_{\text{post}},\theta )\;\Delta r \end{array}$$

where ${\bar{r}}_{pre}=\left(r_{1}+r_{2}\right)∕2$ and Δ*r* = *r*_1_ − *r*_2_ is assumed to be small here. The larger positive $\frac{\partial \Phi }{\partial w}(\overset{¯}{w},{\overset{¯}{r}}_{\text{pre}},r_{\text{post}},\theta )$ is, the more strongly the weights *w*_1_ and *w*_2_ will move apart from each other.

### 3.3. Conditions for joint stability and competition for Hebbian learning with synaptic scaling

In general, the equations for stability and competition may turn out to be quite complex, even for the mean-field dynamical system. The ambition here is to describe the general trends for the influence of τ_Hebb_ and τ_homeo_ on the behavior of the dynamical learning system. To illustrate this, we examine the “simple” case of an arbitrary Hebbian-type learning rule with additional synaptic scaling. Inspired by experimental results (Turrigiano and Nelson, 2000) and used in previous studies (van Rossum et al., 2000; Yger and Harris, 2013; Zenke et al., 2013), synaptic scaling is used as a homeostatic mechanism that increases or decreases homogeneously the synaptic weights in order to reach a given firing rate *r*_target_. In our generic formulation in Equation (2), this is equivalent to including an additive scaling term Γ in the expression of Φ in addition to the Hebbian contribution *H*, while θ tracks the post-synaptic firing rate with a timescale τ_homeo_.

(10) $$\begin{array}{l} \Phi (\bar{w},r_{\text{pre}},r_{\text{post}},\theta )=H(\bar{w},r_{\text{pre}},r_{\text{post}})+\Gamma (\bar{w},\theta )\\ \;\Gamma (\bar{w},\theta )=\alpha \bar{w}(r_{\text{target}}-\theta )\\ \;\Psi (r_{\text{post}})=r_{\text{post}} \end{array}$$

For simplicity, we rewrite the Hebbian contribution using Equation (3) in terms of $\bar{w}$ only: $\tilde{H}\left(\bar{w}\right):=H\left(\bar{w},r_{pre},r_{post}\right)$. This yields the following expression for the Jacobian in Equation (4):

(11) $$\left(\begin{array}{cc} \frac{{\tilde{H}}^{\prime }(\bar{w})+\alpha (r_{\text{target}}-\theta )}{\tau_{\text{Hebb}}} & \frac{-\alpha \bar{w}}{\tau_{\text{Hebb}}}\\ \frac{r_{\text{pre}}}{\tau_{\text{homeo}}} & \frac{-1}{\tau_{\text{homeo}}} \end{array}\right)$$

The equilibrium corresponds to the fixed point(s) where $\dot{\bar{w}}=0$ and $\dot{\theta }=0$, which implies that *r*_post_ = θ and $\tilde{H}\left(\bar{w}\right)=-\alpha \bar{w}\left(r_{target}-\theta \right)$. The trace and determinant of the Jacobian matrix are given by

(12) $$\begin{array}{l} T=\frac{{\tilde{H}}^{\prime }(\bar{w})-\tilde{H}(\bar{w})/\bar{w}}{\tau_{\text{Hebb}}}-\frac{1}{\tau_{\text{homeo}}}\\ D=\frac{-[{\tilde{H}}^{\prime }(\bar{w})-\tilde{H}(\bar{w})/\bar{w}]+\alpha r_{\text{post}}}{\tau_{\text{Hebb}}\tau_{\text{homeo}}} \end{array}$$

As explained above, stability is ensured when the necessary conditions *T* < 0 and 0 < 4*D* < *T*^2^ in Equation (6) are met. These three conditions read.

(13) $${\tilde{H}}^{\prime }(\bar{w})-\frac{\tilde{H}(\bar{w})}{\bar{w}}<\frac{\tau_{\text{Hebb}}}{\tau_{\text{homeo}}}$$

(14) $${\tilde{H}}^{\prime }(\bar{w})-\frac{\tilde{H}(\bar{w})}{\bar{w}}<\alpha r_{\text{post}}$$

(15) $$\alpha r_{\text{post}}<\frac{1}{4}{\left\{\left[{\tilde{H}}^{\prime }(\bar{w})-\frac{\tilde{H}(\bar{w})}{\bar{w}}\right]\sqrt{\frac{\tau_{\text{homeo}}}{\tau_{\text{Hebb}}}}+\sqrt{\frac{\tau_{\text{Hebb}}}{\tau_{\text{homeo}}}}\right\}}^{2}$$

The term ${\tilde{H}}^{\prime }\left(\bar{w}\right)-\tilde{H}\left(\bar{w}\right)∕\bar{w}$ corresponds to the sub/super-linearity of the effective weight update $\tilde{H}$ at the equilibrium $\bar{w}$, including the effects of the neuron model. For the simplest Hebbian rule $H\left(\bar{w},r_{pre},r_{post}\right)=r_{pre}r_{post}$, $\tilde{H}\left(\bar{w}\right)=r_{pre}^{2}\bar{w}$ is linear and we always have $\tilde{H}\left(\bar{w}\right)-\tilde{H}\left(\bar{w}\right)∕\bar{w}=0$. This implies that the first two conditions Equations (13) and (14) are always true, while the third condition Equation (15) reduces to α*r*_post_ < τ_Hebb_ ∕ 4τ_homeo_. For the synaptic scaling mechanism, α should be chosen sufficiently large in order to keep the output rate *r*_post_ close to its target *r*_target_. It follows that the third condition may be violated depending on the details of the parameters, in particular when τ_Hebb_ ≪ τ_homeo_. This corresponds to non-real eigenvalues, synonymous with oscillatory dynamics in the weights.

As a second example related to the BCM rule and triplet-STDP as will be detailed later, when $\tilde{H}$ is a quadratic polynomial in $\bar{w}$ with positive second-order coefficient, we have ${\tilde{H}}^{\prime }\left(\bar{w}\right)-\tilde{H}\left(\bar{w}\right)∕\bar{w}>0$ for large weights. According to Equation (9), a large positive value for ${\tilde{H}}^{\prime }\left(\bar{w}\right)$ implies strong competition as desired. However, the condition for the negativity of the trace in Equation (13) implies that τ_homeo_ should not be much larger than τ_Hebb_, as shown previously (Zenke et al., 2013). Then, assuming Hebbian learning to be relatively fast, Equations (14) and (15) define a limited range for the choice of α, out of which divergence or oscillations may occur. As a conclusion, those stability and competition conditions oppose each other and make the fine tuning of the parameters necessary.

## 4. The family of STDP learning rules

### 4.1. Need for regulation with classical STDP

As a first example of learning rules, we consider the family of STDP rules to illustrate the interplay between Hebbian learning and synaptic scaling. We show that they fall into the mathematical framework developed in Section 3. To start, without any additional homeostatic regulation based on θ, we recall that the convergence of the weight depends on the fixed points of Φ only. The original version of STDP simply describes the effect for pairs of input-output spikes using the well-known temporal window in Figure 1B, which determines the weight update as a function of spike-time difference. All contributions are then summed over time to obtain the total weight update. The net effect denoted by *H* here can be decomposed into two terms, for the neuronal firing rates and covariances, respectively (Kempter et al., 1999; Gilson et al., 2009). In our framework based on the Poisson neuron (see Section 3), this gives the following differential equation for the mean weight $\bar{w}$

(16) $$\begin{array}{l} \dot{\bar{w}}=\frac{1}{\tau_{\text{Hebb}}}H(\bar{w},r_{\text{pre}},r_{\text{post}},c_{\text{pre-post}})\\ \;=\frac{1}{\tau_{\text{Hebb}}}(Ar_{\text{pre}}r_{\text{post}}+Bc_{\text{pre-post}})\\ \;=\frac{1}{\tau_{\text{Hebb}}}(Ar_{\text{pre}}^{2}+Bc_{\text{pre}})\bar{w} \end{array}$$

where the typical area under the curve *A* < 0 corresponds to more LTD than LTP for the rate contribution, while *B* > 0 describes LTP due to the temporal interaction for correlated inputs. The last line is obtained using Equation (3), where the mean weight update can be expressed as a linear function of the weight from a macroscopic point of view. We obtain a first-order polynomial similar to that for classical Hebbian learning, where the coefficient depends on the input correlation. Two behaviors can occur for this system: for sufficiently strong input correlations *c*_pre_, the factor for $\bar{w}$ becomes positive and the fixed point unstable, so positive weights are potentiated in a Hebbian fashion and diverge; otherwise weights are depressed and converge to the fixed point $\bar{w}=0$. For a pool of synapses, competition is ensured provided $\frac{\partial H}{\partial w}=\left(Ar_{pre}^{2}+Bc_{pre}\right)∕\tau_{Hebb}>0$, which occurs for sufficiently strong input correlation here. In that case, the diverging learning dynamics can result in a bimodal distribution when a positive upper bound is set (Kempter et al., 1999; Song and Abbott, 2001).

To change the fixed-point structure and enforce stability, one can add a penalty term on the weight update based on the current value of the weight (Oja, 1982). A usual example found in the literature uses a polynomial in $\bar{w}$, which leads to the following expression for Φ.

(17) $$\dot{\bar{w}}=\frac{1}{\tau_{\text{Hebb}}}(Ar_{\text{pre}}^{2}+Bc_{\text{pre}})\bar{w}\;-\;\alpha {\bar{w}}^{n}$$

The key point here is that ẇ is a first-order polynomial in *w* for classical STDP, so *n* ≥ 2 stabilizes the system (Tetzlaff et al., 2011). Synaptic scaling maintains the synaptic competition while preventing weights from taking too high values, at the cost of not being able to control the post-synaptic firing rate, and without having any relationship to the real homeostatic timescale. Although that previous work studied in depth the interaction of synaptic scaling with more complex Hebbian learning rule, the temporal dynamics when the two processes are not acting on the same timescale is still poorly understood.

### 4.2. Synaptic scaling mechanism targeting a fixed firing rate requires fine tuning

In order to target a fixed firing rate, weight normalization as previously defined is not sufficient. One must add a constraint enforcing the post-synaptic neuron to scale all its input weights such that, on average, a desired firing rate is maintained. Following previous studies (van Rossum et al., 2000; Yger and Harris, 2013; Zenke et al., 2013), it can be implemented by the term Γ as in Equation (10), which depends on the difference between a running estimate of the post-synaptic firing rate and a desired firing rate *r*_target_. The expression for Φ with the STDP contribution *H* and Ψ then read

(18) $$\begin{array}{l} \Phi (\bar{w},r_{\text{post}},c_{\text{pre}-\text{post}},\theta )=H(\bar{w},r_{\text{post}},c_{\text{pre}-\text{post}})\\ \;+\;\alpha \bar{w}(r_{\text{target}}-\theta )\\ \;\Psi (r_{\text{post}})=r_{\text{post}} \end{array}$$

The constant α defines the strength of the homeostasis on the mean weight $\bar{w}$, while τ_homeo_ determines the timescale of the smoothing of the *r*_post_ estimate tracked by θ.

**Figure 2:**
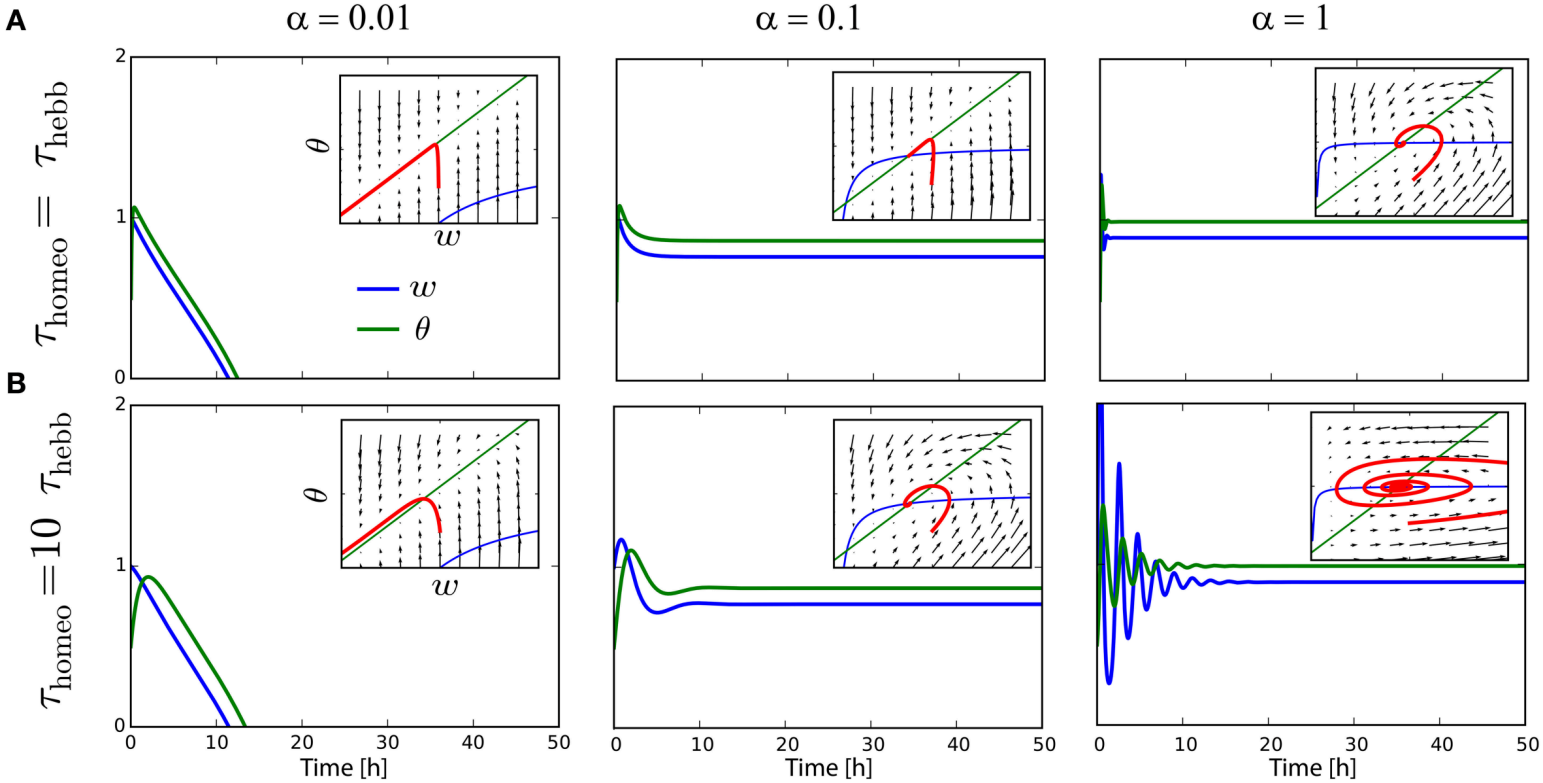
**Interplay between Hebbian and homeostatic timescales for pairwise STDP**. **(A)** Evolution of the weight *w* and the running estimate θ of the post-synaptic firing rate as function of time, for τ_Hebb_ = τ_homeo_ = 10 min and various gain α for the heterosynaptic scaling. Insets shows the trajectory in the phase space (*w*, θ). **(B)** Same as **(A)** with a slower homeostatic scaling: τ_homeo_ = 10 τ_Hebb_ = 100 min.

The analysis in Section 3 states that τ_Hebb_, τ_homeo_ and α must be chosen so as to avoid instability and trivial solutions where all weights become silent. As it has been shown for other learning rules (Cooper et al., 2004; Zenke et al., 2013), the running estimate θ of the post-synaptic firing rate have to be rather fast, otherwise the system is subject to strong oscillations. To illustrate the problem, suppose we have a neurons targeting *r*_target_ = 1 Hz, with *r*_pre_ = 0.9 *Hz, c*_pre_ = 0.1, *A* = −0.1 and *B* = 1 (see Section 4.1). The value of α is varied between 0.01, 0.1, and 1. As we can see on Figure 2A, the convergence to the fixed point can be pretty fast if τ_Hebb_ = τ_homeo_, and if α is strong enough to counterbalanced the Hebbian force that depresses synapses here; see panels with α ≥ 0.1, insets show the trajectory in the phase space $\left(\bar{w},\theta \right)$ as function of time. However, we can see on Figure 2B that when τ_homeo_ ≫ τ_Hebb_, as it is found *in vivo* (Keck et al., 2013), strong oscillations emerge for strong value α = 1. There is a fine tuning required between those two competing forces. To circumvent the problem, the use of a Proportional-Integral (PI) controller was incorporated in some study (van Rossum et al., 2000; Yger and Harris, 2013), but even when it prevents some oscillations from occuring, it does not abolish the requirement that τ_Hebb_ and τ_homeo_ should not be order of magnitudes apart.

### 4.3. Similar stability issues occur for weight-dependent and triplet STDP

The analysis and the observations performed previously can be extended to several STDP-like learning rules. For example, a simple version of the weight-dependent STPD learning rule (van Rossum et al., 2000; Morrison et al., 2007; Gilson and Fukai, 2011) with linearly increasing LTD as a function of the weight and constant LTP gives

(19) $$\begin{array}{l} H(\bar{w},r_{\text{post}},c_{\text{pre-post}})=(A_{+}+A_{-}\bar{w})r_{\text{pre}}r_{\text{post}}\\ \;+\;Bc_{\text{pre}-\text{post}}\\ \;=(A_{+}r_{\text{pre}}^{2}+Bc_{\text{pre}})\bar{w}+A_{-}r_{\text{pre}}^{2}{\bar{w}}^{2} \end{array}$$

**Figure 3:**
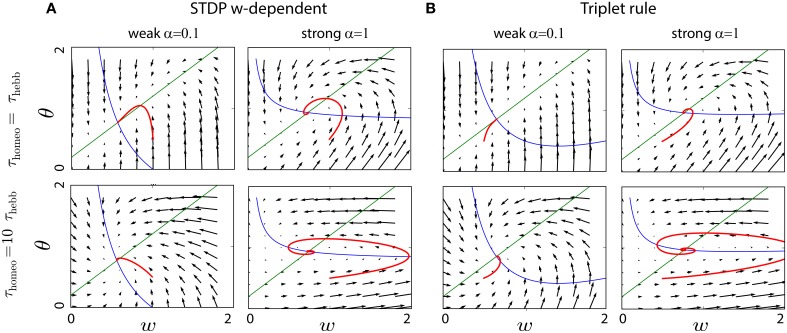
**Interplay between Hebbian and homeostatic timescales for different learning rules and homeostatic forces**. **(A)** Pairwise STDP with weight dependent modification in Equation (19). Left column: convergence in the phase space (*w*, θ) for a fast homeostatic force (τ_Hebb_ = τ_homeo_ = 10 min, upper row), or for a slow homeostatic force (τ_homeo_ = 10τ_Hebb_, lower row). Right column is the same, but with a stronger drive α = 1 for the homeostatic force. **(B)** Same as **(A)** for the triplet learning rule (Pfister and Gerstner, 2006), see Equation (20).

Again Equation (3) was used to obtain the second-order polynomial in $\bar{w}$. In Figure 3B that depicts the convergence of the system in a similar fashion to Figure 2 with typical values for the parameters (*A*_+_ = 0.1, *A*_−_ = −0.3, *B* = 1, *c*_pre_ = 0.1), the convergence is achieved if homeostatic coupling is weak (α = 0.1). However, large oscillations arise for strong coupling (α = 1) and when the ratio between the homeostatic and Hebbian timescales is large.

Likewise, the triplet STDP model (Pfister and Gerstner, 2006) corresponds to

(20) $$\begin{array}{l} H(\bar{w},r_{\text{post}},c_{\text{pre-post}})=(A_{+}r_{\text{post}}+A_{-})r_{\text{pre}}r_{\text{post}}\\ \;+Bc_{\text{pre-post}}\\ \;=(A_{-}r_{\text{pre}}^{2}+Bc_{\text{pre}})\bar{w}+A_{+}r_{\text{pre}}^{3}{\bar{w}}^{2} \end{array}$$

where *A*_+_ > 0, *A*_−_ < 0 for the LTP and LTD rate contributions, respectively, as well as *B* > 0 for the correlation contribution. Again, for standard values of the parameters *A*_+_ = 0.05, *A*_−_ = −*0.2, B* = 1, *c*_pre_ = 0.1 (Pfister and Gerstner, 2006), we see in Figure 3B the same qualitative behavior as with weight-dependent STDP.

The similarity can be explained by the fact that both Equations (19) and (20) are quadratic polynomials in $\bar{w}$. The difference between the two rules lies in the signs of the coefficients. Nevertheless, we have for the scaling term $\Gamma \left(\bar{w},\theta \right)=\alpha \bar{w}\left(r_{target}-\theta \right)≃\alpha r_{target}\bar{w}-\alpha r_{pre}{\bar{w}}^{2}$, where we have used $\theta ≃r_{post}=r_{pre}\bar{w}$. This means that, when Γ overpowers the STDP contribution to enforce stability with a large α, the coefficient for $\bar{w}$ in Φ is negative in both cases. It ensures stability, but generates similar oscillations for large τ_homeo_. The intuitive explanation is that large values for τ_homeo_ cause the gradient to have a strong horizontal component in the phase space $\left(\bar{w},\theta \right)$ of Figure 3, which often implies oscillations around the fixed point.

### 4.4. Trade-off between stability and competition

**Figure 4:**
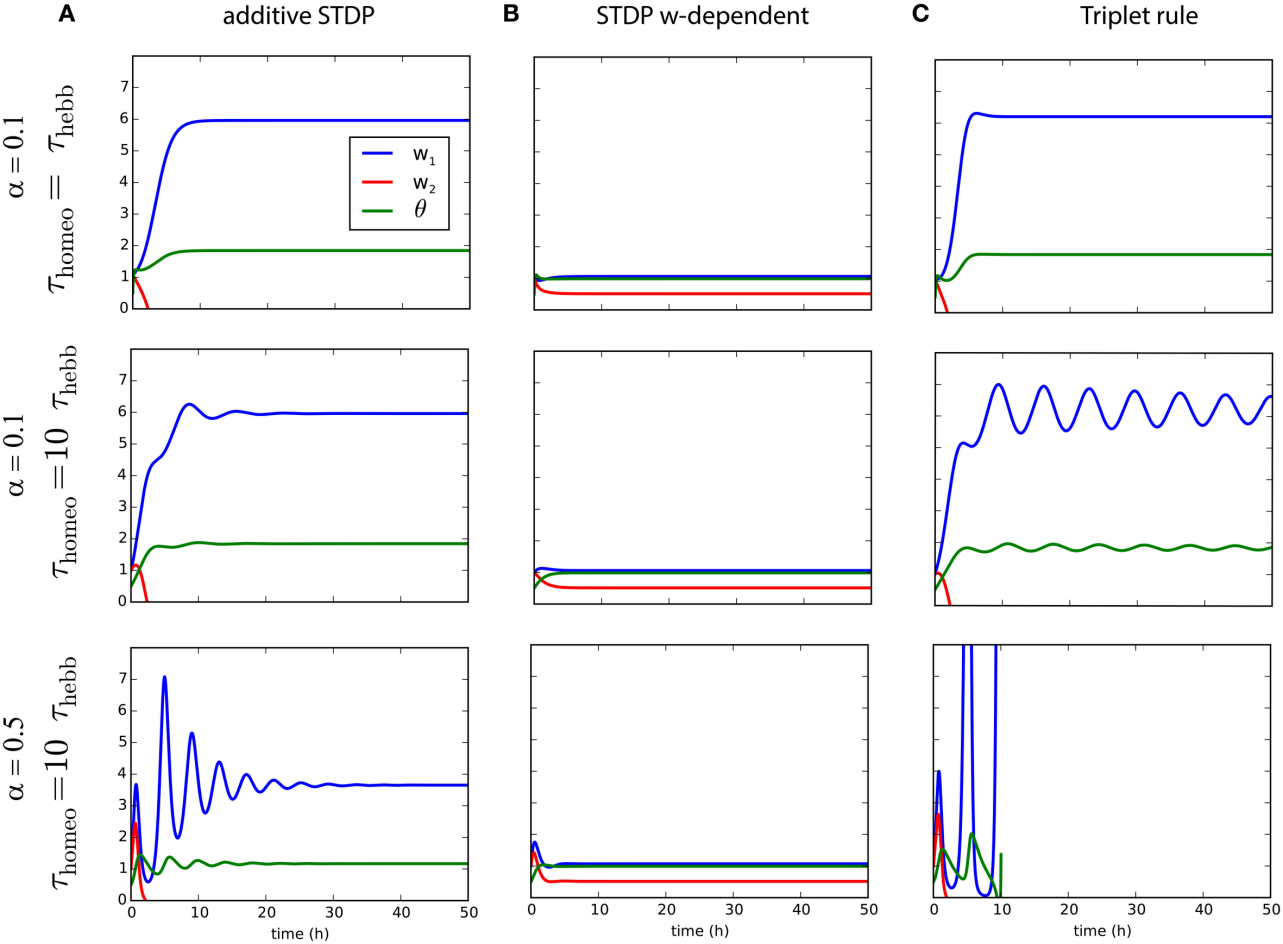
**Competition for several plasticity rules with different timescales for Hebbian and homeostatic forces**. **(A)** Pairwise STDP with weight-independent update. Convergence of two synaptic weights *w*_1∕2_ with different correlation inputs and the estimate of the post-synaptic firing rate, θ as function of time, for a fast homeostatic force (τ_Hebb_ = τ_homeo_ = 10 min, top row), for a slow homeostatic force (τ_homeo_ = 10τ_Hebb_, middle row), or for a slow and stronger homeostatic force (α = 0.5). **(B)** Same as A for the weight-dependent STDP learning rule (van Rossum et al., 2000). **(C)** Same as **(A)** for triplet learning rule (Pfister and Gerstner, 2006).

While we analyzed the dynamical behavior of the learning rules for the mean weight to assess their implications for stability, we now examine the situation for two inputs in order to study how competition can be affected by this interaction between homeostatic and Hebbian learning. This yields an extra differential equation as explained in 3.2. Figure 4 illustrates the evolution of the weights *w*_1_ and *w*_2_, as well as θ, for the three learning rules previously mentioned combined with synaptic scaling, and show how competition can take place. We consider two input pathways with the same input rates *r*_1∕2_, but different levels of correlation: $c_{pre}^{1}=0.1$ and $c_{pre}^{2}=0.05$. The homeostatic mechanism targets the fixed firing rate *r*_target_ = 2 Hz. As shown in Figures 4A,C, strong competition is observed for both classical STDP and triplet STDP, leading to *w*_2_ = 0 for the pathway with weaker correlation *c*_2_ < *c*_1_ (Kempter et al., 1999; van Rossum et al., 2000; Song and Abbott, 2001). For weight-dependent STDP, the competition is much weaker in Figure 4B. Nevertheless, in all cases, increasing the ratio τ_homeo_ ∕ τ_Hebb_ introduces oscillations of the weights during competition, exactly as previously observed for the mean weight $\bar{w}$. We also see that an increased strength for the homeostatic force (α = 0.5 in the bottom row of Figure 4) does not solve the stability issue when τ_homeo_ ≫ τ_Hebb_, but causes larger fluctuations.

## 5. Metaplastic learning rules

The previous section showed the common trend for STDP learning rules paired with synaptic scaling targeting a desired firing rate: a large time constant to estimate the post-synaptic firing rate gives rise to instability or potentially large oscillations in the weights. Now we examine a second category of stabilizing mechanisms, where the homeostatic mechanism is implemented directly in the metaplastic learning rule; see Yeung et al. (2004) for an example for calcium-based regulation. Metaplasticity is often used to enforce a homeostatic behavior on the neural system and we will stick to this function here. Without loss of generality, we ignore correlations in the learning rules and focus on rate-based rules.

### 5.1. The bienenstock-cooper-munro (BCM) learning rule

In order to extend rules based on correlations of rates (Oja, 1982) and approach the problem of synaptic competition via weight normalization, Bienenstock et al. (1982) designed a model of synaptic plasticity that was able to reproduce phenomenologically several observations made *in vivo*. Their so-called BCM rule is a physical theory of learning in the visual cortex; see Cooper and Bear (2012) for a review. The mechanism consists in an efficient way to balance and regulate the amount of plasticity according to past activity by means of a heterosynaptic process.

Practically, a sliding threshold determines the boundary between LTP above and LTD below, and evolves according to the square of the postsynaptic firing rate (Bienenstock et al., 1982). In our formalism, this can be taken care of by a temporal tracking of $r_{post}^{2}$ using θ as the threshold variable with τ_homeo_ ≫ τ_Hebb_, such that $\theta ≃\langle r_{post}^{2}\rangle$ with the angular brackets indicating the average over the randomness. The expression for Φ is a second-order polynomial in *r*_post_ (Bienenstock et al., 1982), which finally gives

$$\begin{array}{l} \Phi (\bar{w},r_{\text{post}},\theta )=r_{\text{pre}}r_{\text{post}}(r_{\text{post}}-\theta )\\ \;\Psi (r_{\text{post}})=r_{\text{post}}^{2} \end{array}$$

Here Φ has a similar form to that for the triplet rule in Equation (20), but the boundary between potentiation and depression is now given by θ.

**Figure 5:**
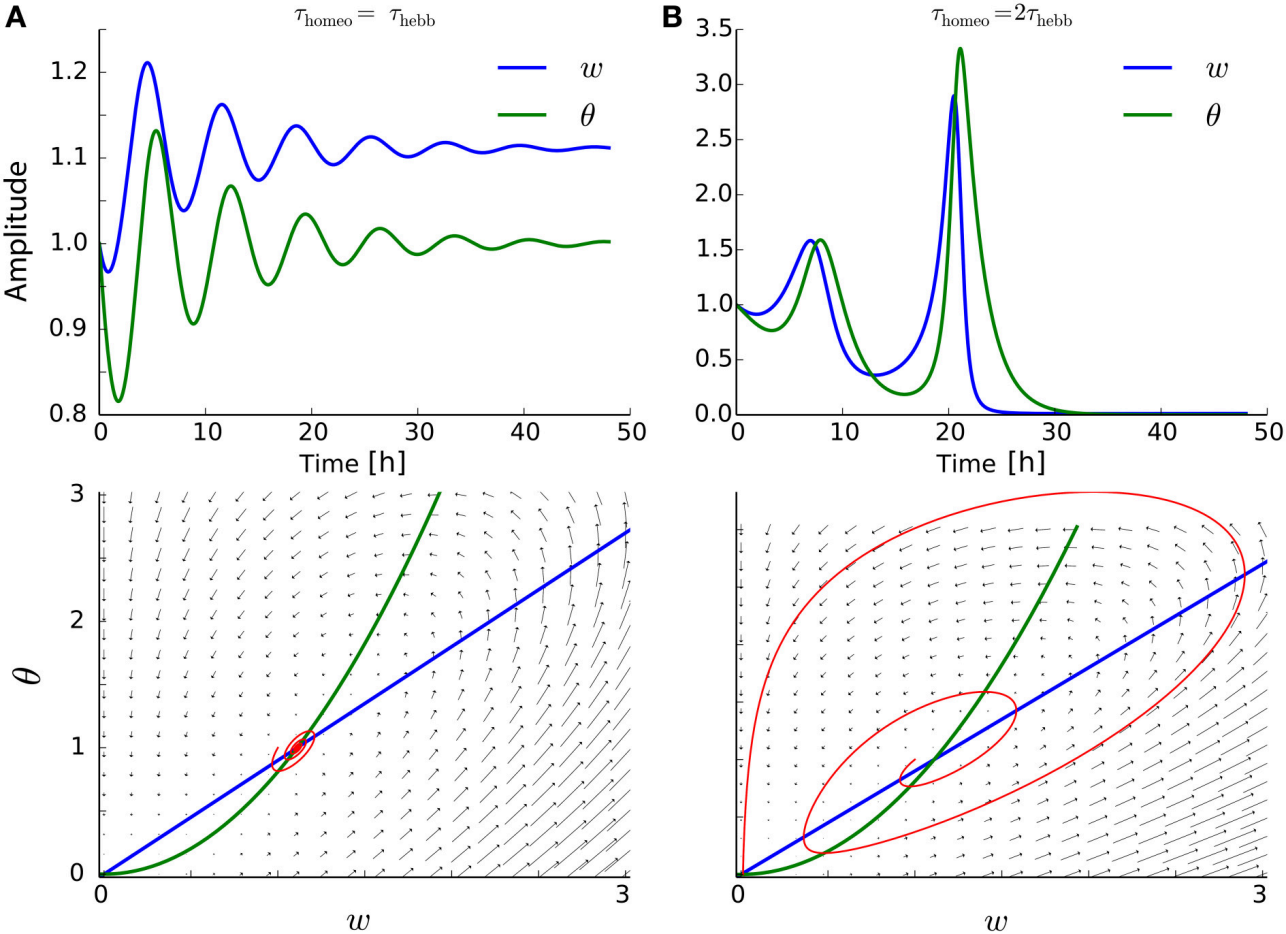
**Interplay between Hebbian and homeostatic timescales for the BCM learning rule**. **(A)** Upper row: evolution of the weight and the running estimate Ψ of the post-synaptic firing rate as function of time, for τ_Hebb_ = τ_homeo_ = 10 min. Lower row: same but in the phase space (*w*, θ). **(B)** Same as **(A)** with τ_homeo_ = 2τ_Hebb_.

It is known that the BCM formalism can be subject to strong oscillations, when the timescales for the two differential equations are too far apart (Cooper et al., 2004; Toyoizumi et al., 2014). In Figure 5A, even when τ_Hebb_ = τ_homeo_, weight oscillations are present. Moreover, for a slightly larger ratio τ_Hebb_ ∕ τ_homeo_, the oscillations can destroy the convergence of the system when the weights hit the lower bound 0, as illustrated in Figure 5B.

### 5.2. Modulation of STDP depending on the post-synaptic firing rate

In order to stabilize the triplet STDP rule (Pfister and Gerstner, 2006) in recurrent networks, further studies (Clopath et al., 2010; Zenke et al., 2013) scaled the amount of LTD in terms of a smoothed average of the firing of the post-synaptic neuron. This modulation of LTD actually brings the triplet STDP rule closer to the BCM rule, by implementing a regulation of the threshold between effective LTP and LTD. In our formalism, the rule used by Zenke et al. (2013) can be implemented for rates as

$$\begin{array}{l} \Phi (\bar{w},\;r_{\text{post}},\;\theta )=r_{\text{pre}}r_{\text{post}}(A_{+}r_{\text{post}}+A_{-}\theta^{2}/r_{\text{target}})\\ \;\Psi (r_{\text{post}})=r_{\text{post}} \end{array}$$

Note that the difference here compared to BCM is that θ tracks *r*_post_ and not $r_{post}^{2}$, and the limit between depression and potentiation is related to θ^2^. As in Equation (20), we have *A*_+_ > 0 and *A*_−_ < 0.

**Figure 6:**
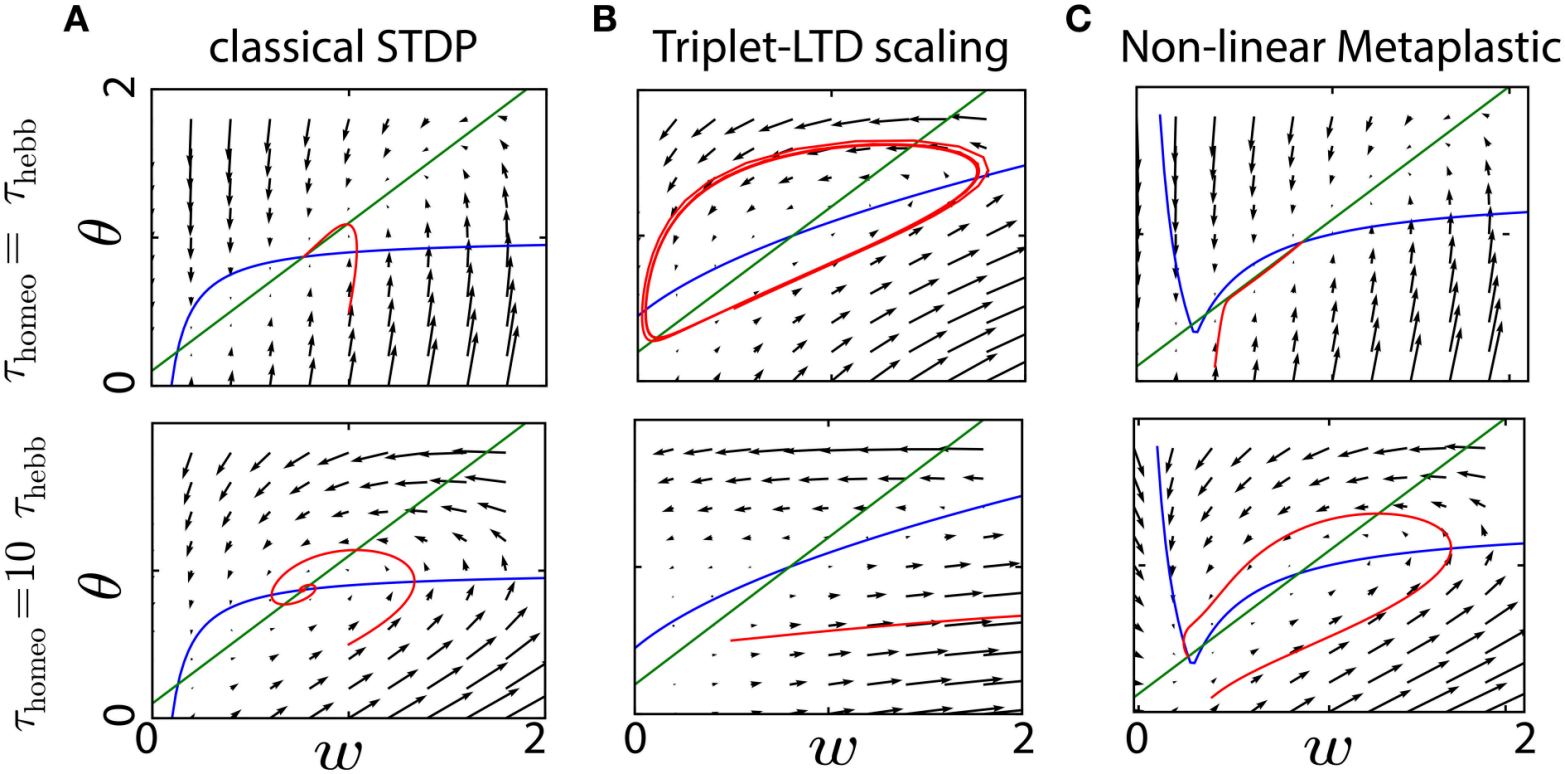
**Convergence of metaplastic learning rules**. **(A)** Convergence in the phase space (*w*, θ) of a classical STDP plasticity, either for a fast homeostatic time constant (τ_homeo_ = τ_Hebb_, upper row) or for a slow one (τ_homeo_ = 10 τ_Hebb_, lower row). **(B)** Same as **(A)** for a metaplastic learning rule combining the triplet learning rule and scaling of the LTD term (Zenke et al., 2013). **(C)** Same as **(A)** for a non-linear metaplastic learning rule including thresholds (El Boustani et al., 2012).

Figure 6 compares the evolution for this metaplastic triplet STDP rule with classical STDP combined with synaptic scaling: we can clearly see that the resulting dynamics is strongly affected by the ratio between Hebbian and homeostatic time constants in both cases. The trajectories of *w* and θ in the same phase space as before show several types of instability, from weight (and rate) explosion for slow tracking with large τ_homeo_ (Zenke et al., 2013) to oscillations when τ_homeo_ = τ_Hebb_. As before, slow tracking yields a gradient with a strong horizontal component, hence oscillations. The limit cycle in the top panel of Figure 6B only happens for some limited range of the parameters, but this illustrates the severe instability issues even for the simple dynamical system considered here.

### 5.3. Non-linearly gated STDP rules

Another direction of research (Senn et al., 2001; El Boustani et al., 2012) introduced non-linearity in the effect of the Hebbian term, by turning it on and off depending on the past pre- and post-synaptic activity of the neuron. Taking a simplified version with a similar mechanism for both LTP and LTD, we consider

(21) $$\begin{array}{l} \Phi (\bar{w},\;r_{\text{post}},\;\theta )=\|r_{\text{pre}}r_{\text{post}}\\ \;-f_{+}(\theta ){\|}_{+}-\|r_{\text{pre}}r_{\text{post}}-f_{-}(\theta ){\|}_{+}\\ \;\Psi (r_{\text{post}})=r_{\text{pre}}r_{\text{post}} \end{array}$$

where ∥*x*∥_+_ is a non linear function equal to *x* if *x* > 0 and 0 otherwise. Now Ψ is such that θ embodies a smoothed average of the pre-post correlations with the time constant τ_homeo_. When instantaneous correlations are higher than thresholds *f*_±_(θ), for potentiation or depression respectively, plasticity effectively occurs. In the general case, *f*_±_ could be any non-linear functions, and do not even need to rely on the same timescales (El Boustani et al., 2012). The simulations in Figure 6C correspond the simple case where *f*_±_(θ) = *a*_±_ = ±0.4 are constant. The problem with those non-linearities is that it becomes hard to perform an mathematical analysis of the equilibrium. As with other rules, we observe the same effect of a large τ_homeo_ on the gradient and the same qualitative conclusion that slow tracking implies the slow convergence of the system.

### 5.4. Toward more complex models

The stability problem arises because, at the equilibrium state, the Hebbian and homeostatic mechanisms compete to balance each other, but they do not act on the same timescale. As pointed out recently (Toyoizumi et al., 2014), a solution can be found when considering that both do not interact linearly, i.e., summing their effects at the synapses, but rather work in a multiplicative manner to determine the synaptic weight. To be more precise, the model developed by Toyoizumi et al. (2014) can be integrated within our framework modulo a slightly more generic formulation for the equation in θ. The model states that *w* = ρ*H*, where those two quantities are governed by the following system of differential equations.

$$\begin{array}{l} \dot{\rho }=\frac{1}{\tau_{\text{hebb}}}[(\rho_{\text{max}}-\rho )\|r_{\text{pre}}r_{\text{post}}-A_{+}{\|}_{+}\\ \;-(\rho -\rho_{\text{min}})\|A_{-}-r_{\text{pre}}r_{\text{post}}{\|}_{+}]\\ \dot{H}=\frac{1}{\tau_{\text{homeo}}}H(1-r_{\text{post}}) \end{array}$$

Even if the lower and upper weight bounds ρ_min_ and ρ_max_ depend on *H*, the model can be written in a generalized version of Equation (2), using $ẇ=H\dot{\rho }+Ḣ\rho$ with simply θ = *H*. The final expression resembles non-linearly gated plasticity with an additional synaptic scaling, but involves further refinements compared to Equation (21).

## 6. Discussion and perspectives

In this paper, we have reviewed various homeostatic mechanisms that are used in recent state-of-the-art plasticity models to regulate Hebbian-type learning. We have focused on two main categories of models: (1) homeostatic synaptic scaling as an independent process that competes with the Hebbian force via an additive term, and (2) metaplastic rules, for which the Hebbian contribution is modulated in an homeostatic fashion. In both cases, the regulation is performed via an estimate of the neural activity (often the post-synaptic firing rate *r*_post_) smoothed with a timescale τ_homeo_, whereas the Hebbian update corresponds to another timescale τ_Hebb_. We have shown for most models that, when τ_homeo_ ≫ τ_Hebb_, undesired behaviors such as oscillations in the synaptic weights occur, in particular in the case where the homeostatic force is strong. Moreover, competition and stability correspond to conflicting constraints on the parameters, which requires fine-tuning. There is thus a trade-off between the strength of the homeostatic regulation that must compete with the Hebbian drive without perturbing the convergence to a fixed point for the weights. Stability in the weights at a macroscopic level is necessary to ensure stability of the neural functions; note that we have not considered noise in the dynamics of individual weights here, but rather their mean for given pathways.

This constraint on the timescales τ_Hebb_ and τ_homeo_ is problematic in regards of available experimental data, as many of them point to slow homeostatic processes (Turrigiano et al., 1998) in comparison with Hebbian processes for which typically τ_Hebb_ ≃ 10 min. Other models not considered here exhibit similar behavior, for example a homeostatic regulation obtained via intrinsic plasticity (see Zheng et al., 2013) for an example based on spike-threshold adaptation. As a conclusion, the control of the firing rate of the post-synaptic neuron should be taken care of by a mechanisms at a fast timescale, say few minutes at the maximum. Conversely, we point out that homeostatic mechanisms operating on a much slower timescale should be related to other functions than maintaining the neural activity in a given range.

This claim is supported by several experimental and theoretical findings. Spiking activity of neurons *in vivo* is known to be sparse and highly irregular. Most V1 neurons display Poissonnian or supra Poisson spike-count variability in response to low dimensional stimuli such as bars and gratings (Dean, 1981). Even *in vitro*, they fire as Poisson sources, irregularly, with a coefficient of variation for their inter-spike intervals close to 1 (Nawrot et al., 2008). The origin of this irregular activity observed in the sub-threshold voltage and/or in spiking activity is linked to synaptic activity (Paré et al., 1998; Destexhe and Paré, 1999), and because it has been observed experimentally that excitatory and inhibitory conductances are closely balanced (Froemke et al., 2007; Okun and Lampl, 2008), such a fine balance has to be maintained by the system (Renart et al., 2010). Therefore, there is a crucial need for compensatory mechanisms that may interfere or act in concert with Hebbian learning to not only keep the neuron's firing rate within a certain range, but also guarantee this balance (Vogels et al., 2011), or the irregularity of the spiking discharge (Pozzorini et al., 2013). Weight normalization has also been studied in depth in the context of emergence of ocular dominance in order to adjust the competition between synaptic pathways, switching from winner-take-all to winner-share-all behaviors for example (Miller, 1996).

We should discuss several limitations of our study related to the proposed mathematical framework. We have focused on very simple and canonical models of synaptic plasticity, ignoring the fine morphological structure of the neurons. It was shown that the shape of the temporal learning window represented in Figure 1B depends on the synaptic position on the dendritic tree (Letzkus et al., 2006; Kampa et al., 2007). More importantly, homeostatic regulation or plasticity thresholds exhibit variability and affect predominantly neighboring synapses *in vivo* (Harvey and Svoboda, 2007). Therefore, we only address the temporal crosstalk between Hebbian and homeostatic plasticity at the largest scale and the question of defining the spatial extent for heterosynaptic mechanisms remains open. Nevertheless, we expect our conclusions to hold locally for groups of synapses that can be isolated and experience homogeneous processes.

In the general dynamical system in Equations (2) considered here, the timescales are explicitly defined via τ_Hebb_ and τ_homeo_. In more complex dynamical systems involving noise and attractors, implicit time constants can emerge in a population of synapses (Tetzlaff et al., 2013). Usually, they are slow time constants though, and cannot be used for fast control of the rate, but rather to implement long-lasting memory patterns in the synaptic weights. Another limitation of our conclusions is that we only consider a feedforward model. To extend those to networks with plastic recurrent connections, the mathematical formalism should be modified to account for the case of synapses with the same pre- and post-synaptic firing rates *r*_post_ = *r*_pre_ = *r*_rec_, and likewise the correlations *c*_pre−post_ = *c*_rec_. Those quantities follow the consistency equations.

$$r_{\text{rec}}=\frac{r_{\text{in}}}{1-w}\text{ and }c_{\text{rec}}=\frac{c_{\text{in}}}{{\left(1-w\right)}^{2}}$$

A similar analysis can be done to predict the behavior of learning rules and compare them. The difference compared to the feedforward case is that rates and correlations contributions to the weight updates are not of the same order. This implies that oscillations or other instability effects induced by spike synchrony are more likely to be amplified in recurrent networks than those due to firing rates. It remains that stability can be studied similarly via the Jacobian matrix. Note also that noise in firing and learning dynamics, as well as heterogeneity in neuron and network parameters, may help to prevent “pathological” weight trajectories such as limit cycles, as they smooth the dynamical landscape and degenerate too stereotypical situations.

**Figure 7:**
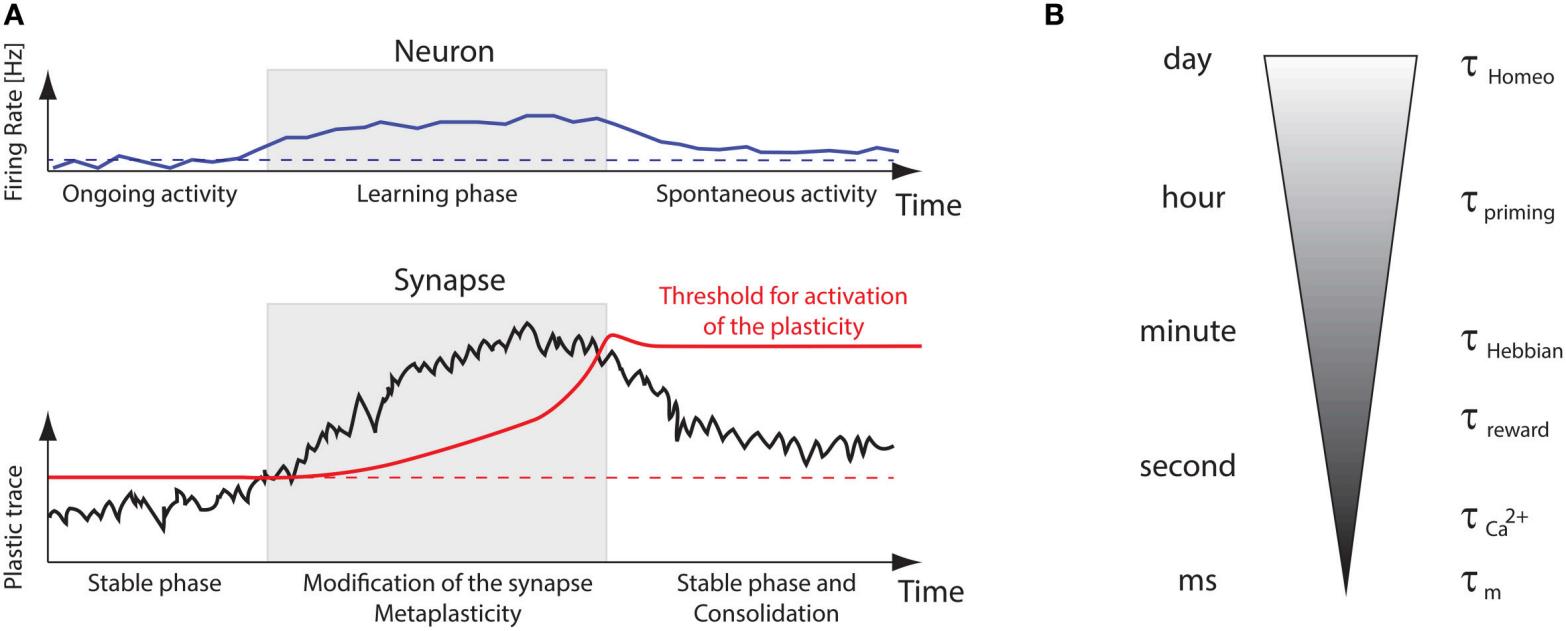
**Memory retention problem and timescales**. **(A)** Illustration of metaplastic thresholds stabilizing the learning. Synapses are stable at in the ongoing regime, then a “plasticity trace” builds up during presentation of new sensory inputs, but this will eventually be stopped by a sliding activation threshold, allowing the synapse to adapt to those novel stimuli. **(B)** Illustration of the multiple timescales involved in plasticity, from the membrane time constant τ_m_ to the homeostatic one τ_homeo_, ranging from ms to days.

Beyond those technical details, the puzzling question with plasticity is how synapses can store relevant information while neurons are constantly bombarded by spiking activity *in vivo*. This ongoing input stimulation is quite often considered to be noise in models, which impairs stability of dynamical systems over long time-scales. Although this issue has been addressed theoretically for various models (Clopath et al., 2008; Billings and van Rossum, 2009; Gilson and Fukai, 2011; Tetzlaff et al., 2013; Zenke et al., 2013), it suggests that additional timescales are necessary to properly combine short-term and long-term properties such that the system learns fast and slowly forgets. Figure 7A recapitulates several timescales involved in learning and memory. In essence, for the neural system to retain memories, synaptic plasticity should only be turned on by metaplasticity when “new” incoming stimuli impinge neurons. Once this novelty has been learnt, metaplasticity should stop synaptic changes. Then a selection process should trim all newly formed memories to keep only appropriate ones (Frey and Morris, 1997). This is illustrated in Figure 7B, where several interleaved timescales interact to bridge all mechanisms, from the effective membrane time constant τ_m_ (order of ms) that interacts with STDP to the homeostatic time constants τ_homeo_, which can range from hours to days (Turrigiano et al., 1998; Turrigiano and Nelson, 2004). Calcium signals can act as activity buffers at a timescale $\tau_{Ca^{2+}}$ (Artola et al., 1990; Shouval et al., 2002; Yeung et al., 2004; Graupner and Brunel, 2012), whereas reward signals or neuromodulation would affect plasticity at a larger timescale τ_reward_ (Izhikevich, 2007), comparable to the one observed for Hebbian changes (τ_Hebbian_). There is also evidence for a control of intrinsic excitability, synaptic scaling at the post-synaptic density, adaptation of the pre-synaptic neurotransmitter release (Davis, 2006). As most models incorporate only a few of those at a time, we stress the need for a better understanding of the complex interactions that may arise when bringing together those experimentally observed mechanisms.

### Conflict of interest statement

The authors declare that the research was conducted in the absence of any commercial or financial relationships that could be construed as a potential conflict of interest.
