# Addendum: Models of Metaplasticity: A Review of Concepts

**DOI:** 10.3389/fncom.2016.00004

**Published:** 2016-01-28

**Authors:** Pierre Yger, Matthieu Gilson

**Affiliations:** ^1^Sorbonne Université, Université Pierre et Marie Curie Univ Paris06 UMRS968 Paris, France; ^2^Institut de la Vision, Institut National de la Santé et de la Recherche Médicale, U968, Centre National de la Recherche Scientifique, UMR7210 Paris, France; ^3^Computational Neuroscience Group, DTIC, Universitat Pompeu Fabra Barcelona, Spain

**Keywords:** synaptic plasticity, metaplasticity, Hebbian learning, homeostasis, STDP

## Author contributions

All authors listed, have made substantial, direct and intellectual contribution to the work, and approved it for publication.

### Conflict of interest statement

The authors declare that the research was conducted in the absence of any commercial or financial relationships that could be construed as a potential conflict of interest.

